# *Lawsonella clevelandensis* is a rare cause of infected chronic contained rupture of abdominal aortic aneurysm

**DOI:** 10.1099/acmi.0.000183

**Published:** 2020-11-25

**Authors:** Wissam Ahmed, Simon Dewar, Robin Williams, Gerard Stansby, Kathryn Harris, Daniel Weiand

**Affiliations:** ^1^​ Freeman Hospital, Freeman Rd, High Heaton, Newcastle upon Tyne NE7 7DN, UK; ^2^​ Great Ormond Street Hospital, Great Ormond St., Holborn, London WC1N 3JH, UK

**Keywords:** 16S rRNA PCR, abdominal aortic aneurysm, co-amoxiclav, fastidious, Gram-positive bacillus, intra-abdominal infection, *Lawsonella clevelandensis*, partially acid fast

## Abstract

*
Lawsonella clevelandensis* is an anaerobic, partially acid-fast, Gram-positive bacillus associated with abscess formation. We present the case of a 70-year-old male with chronic contained rupture of abdominal aortic aneurysm (CCR-AAA) complicated by intra-abdominal abscess formation. An abdominal computed tomography scan revealed a rim-enhancing retroperitoneal collection tracking into the subcutaneous layer of the left flank and buttock, suggestive of CCR-AAA with infected haematoma. He underwent ultrasound-guided needle aspiration of the intra-abdominal collection. Conventional culture techniques failed to isolate *L. clevelandensis *, and the diagnosis was only confirmed by means of 16S rRNA PCR. The patient underwent branched endovascular repair of his aneurysm, and was commenced on treatment with co-amoxiclav, resulting in significant reduction in the size of the infected collection. This is only the second reported case of infection with *L. clevelandensis* in the UK, and the first reported case of this organism causing infected CCR-AAA.

## Introduction

*Lawsonella clevelandensis* is a Gram-positive, partially acid-fast, non-spore-forming, anaerobic, catalase-positive, pleomorphic bacterium, considered a new species within the genus in the suborder *Corynebacterineae* [1, 2]. To date, there have been only seven reported cases of this organism causing infection, having been detected in abdominal [3, 4], spinal [2, 5], liver [2] and breast abscesses [2, 6]. In most cases, *L. clevelandensis* was isolated following prolonged enrichment culture under strict anaerobic atmospheric conditions (mean 7.4 days, range 4–13 days of incubation), but molecular techniques were sometimes required to detect the organism. We present a case of *L. clevelandensis* causing infected chronic contained rupture of abdominal aortic aneurysm (CCR-AAA).

## Case report

This case involves a 70-year-old male under surveillance for an AAA which was below the normal size threshold for treatment. He had a background of hypertension, asthma, osteoarthritis, transient ischaemic attacks and a ventriculo-peritoneal shunt for an intracranial cyst.

In December 2017, he presented to the emergency department at his local hospital, with a 10-day history of feeling unwell and having noticed left lumbar para-spinal swelling and also left gluteal swelling, which were painful to touch. Four days before admission, the patient consulted his general practitioner (GP) and was started on a course of oral clindamycin for probable soft tissue abscess. Between consulting his GP and admission to hospital, the swellings had increased in size and the pain had increased, which limited his mobility. He denied fever but had noticed marked weight loss over 3–4 weeks with reduced appetite. There was no history of trauma or injury.

### Diagnosis

**Fig. 1.**
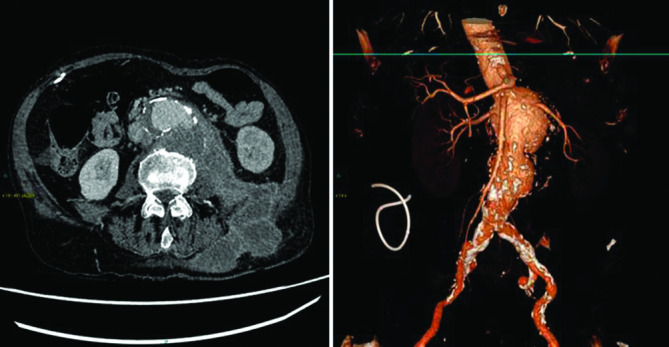
Axial CT and volume rendering technique (VRT) showing ruptured peri-visceral AAA with associated retroperitoneal collection tracking into the subcutaneous layer and showing rim enhancement.

Initial investigations showed a raised white cell count (WCC) of 12.2×10^9^ l^–1^ with a normal neutrophil count of 9.07×10^9^ l^–1^, high C-reactive protein (CRP) levels of 267 mg l^−1^ and elevated platelet count of 674×10^9^ l^–1^. He underwent an abdominal computed tomography (CT) scan which showed an 8 cm peri-visceral AAA, with the partially thrombosed sac in direct communication with a psoas collection tracking into the subcutaneous layer of the left flank and buttock (Fig. 1). The rim-enhancement of the retroperitoneal haematoma was typical of chronic infection. A diagnosis of likely CCR-AAA with infected haematoma was made as the patient met most of the diagnostic criteria for it [7].

He was transferred to the regional specialist vascular service where he underwent ultrasound (US)-guided needle aspiration of the intra-abdominal collection. Although this yielded a small amount of old blood only, and no frank purulent material, the clinical suspicion of infection was high and no specimen was sent for cytological or histopathological analysis.

A Gram stain of the needle aspirate was performed and, although no organisms were seen, numerous white blood cells were seen. No organisms were isolated by means of conventional direct and enrichment culture in a UKAS-accredited microbiology laboratory, accredited to the internationally recognized standard ISO 15189 Medical Laboratories. The specimen was inoculated onto anaerobic blood agar, CLED, chocolate and Campylobacter selective agar. Enrichment culture was performed using Robertson’s broth which, at 48 h of incubation, was subcultured onto anaerobic blood agar. All anaerobic blood agar plates were incubated for 10 days, under strict anaerobic conditions (Whitley MG1000 anaerobic work station), at 35°C, in sterile zip-lock bags [1]. Extended fungal culture on sabouraud agar was negative at 3 weeks.

The sample was also sent to the Microbiology Department at Great Ormond Street Hospital for analysis by broad-range 16S rRNA PCR. This assay is a UKAS-accredited, routine diagnostic service provided by this laboratory. Briefly, the sample was mechanically lysed using bead-beating and DNA was extracted using the Qiasymphony DSP virus/pathogen Mini kit (Qiagen). 16S rRNA gene PCR was performed with published primers and as previously described [8, 9]. The amplicon generated was directly analysed using chain-termination (Sanger) sequencing and the organism present was identified as *L. clevelandensis* by blast analysis against the GenBank database (https://blast.ncbi.nlm.nih.gov) [10].

### Treatment

**Fig. 2.**
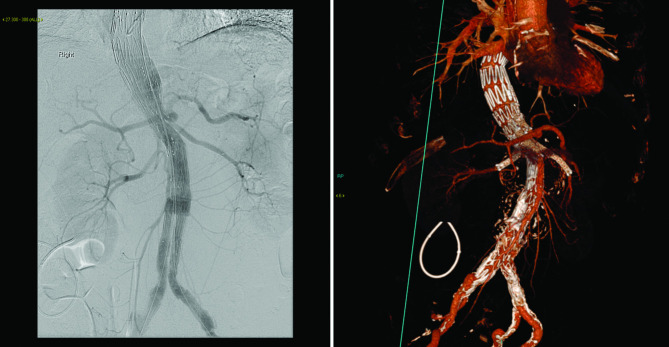
Angiogram and CT and VRT showing branched endovascular repair.

Five days following admission, he underwent endovascular repair of his AAA, which involved the visceral and renal segment of the aorta, following extensive discussion at the vascular surgery multi-disciplinary team meeting. A Cook Medical T-branch endovascular stent-graft was implanted via bilateral common femoral artery access (Fig. 2). Branches to his coeliac, superior mesenteric and both renal arteries were completed from a left subclavian artery access. The stent-graft excluded the ruptured aneurysm and successfully maintained perfusion to the visceral, renal and iliac arteries.

With regard to antimicrobial therapy, on admission he was commenced on a combination of teicoplanin and metronidazole. He remained on teicoplanin until detection of *L. clevelandensis* by means of 16S rRNA PCR, 9 days post-operatively, when treatment was switched to oral co-amoxiclav 625 mg monotherapy to be taken three times a day. Upon discharge from the hospital, 12 days after admission, the plan was for long-term antibiotic therapy with regular follow-up in the vascular clinic, and clinical correlation of follow-up abdominal CT scans. At this stage, the WCC had decreased to 9.6×10^9^ l^–1^, and CRP had decreased to 32 mg l^−1^.

### Outcome and follow up

One month after discharge, the patient again presented to his local hospital feeling unwell and complaining of nausea, vomiting and intermittent fevers. Initial investigations showed high WCC of 17.3×10^9^ l^–1^, high CRP levels of 185 mg l^−1^ and evidence of acute kidney injury, with an elevated creatinine level of 350 µmol l^–1^. A CT angiogram confirmed occlusion of the renal artery branches of the aortic stent-graft. In comparing the CT scans done at the time of his initial presentation and following re-admission, there was a substantial reduction in the size of the retroperitoneal collection. As only a small volume of material had been aspirated from his large collection, which remained largely undrained, the significant reduction in the size of the collection was predominantly due to the treatment with co-amoxiclav. He had an urgent US scan of his kidneys which showed no renal artery blood flow. He was transferred to the renal unit where he underwent urgent haemodialysis. Based on the patient’s comorbidities, recent major operation and declining renal function, further haemodialysis was deemed inappropriate. He was offered best supportive care and died 8 days after re-admission.

## Discussion

Our patient presented with unusual pathology. Most ruptured AAAs are fatal; it is rare to present with CCR-AAA as it constitutes only 4 % of all cases of ruptured AAA [7]. It is even more unusual for the patient to survive long enough for the haematoma to become infected. The CT imaging appearances were strongly suggestive of an infective process, and hence the decision to perform needle aspiration of the collection. When the initial aspirate did not look purulent, we chose not to drain the collection due to the risk of introducing infection and potentially de-tamponading a ruptured AAA.

The choice of technique to repair infected aortas is controversial. Open repair with aortic ligation or replacement with autologous vein has long been considered the gold standard but more recent national registry data from Sweden have suggested better short-term and equivalent long-term mortality with endovascular repair despite the use of prosthetic material [11]. For this patient, endovascular repair was preferred because open repair would have involved clamping of the supra-coeliac aorta and bypass to all visceral and renal arteries with an operative mortality estimated to be significantly greater than 50 %. The national vascular registry report (www.vsqip.org.uk/reports/2018-annual-report) estimates 18.3 % operative mortality for elective supra-renal repairs.

In all previously reported cases of infection with *L. clevelandensis *, management has involved abscess drainage or aspiration with concomitant antimicrobial therapy. In our case, treatment with co-amoxiclav appears to have had a positive influence on the size of the infected retroperitoneal collection.

Menezes *et al*. [6] and Chudy-Onwugaje *et al*. [4] both commenced co-amoxiclav with reported complete resolution of the infection.

In other reported cases, due to the complex nature of the patients’ conditions, or the presence of other organisms, other antimicrobials were administered and often changed during the course of therapy. For example, Navas *et al*. [3] reported a case of *L. clevelandensis* isolated from of an abdominal wall abscess in a 64-year-old Caucasian diabetic male, after he had undergone distal pancreatectomy and splenectomy for resection of a pancreatic neuroendocrine tumour. This patient was empirically commenced on ciprofloxacin and metronidazole, which was later switched to vancomycin, meropenem and fluconazole. The patient remained unwell and was switched to tigecycline, which was later changed to ertapenem. Trimethoprim-sulfamethoxazole was added to treat co-infection with *Stenotrophomonas* species.

As a result of the microscopic similarity between *L. clevelandensis* and *Nocardia* species, previous cases have also been treated with trimethoprim/sulfamethoxazole or meropenem.

Kumaria *et al*. [5] presented a case of subdural empyema caused by *L. clevelandensis *. They treated their patient with intravenous cefuroxime and metronidazole for 2 weeks, followed by oral co-amoxiclav. The abscess recurred and the patient was subsequently commenced on intravenous cefuroxime for 5 days followed by oral linezolid for 4 weeks, which led to complete resolution of the infection.
